# A tactile twist: decoding the phenomena of mechanical itch and alloknesis

**DOI:** 10.3389/fnmol.2023.1278151

**Published:** 2023-09-12

**Authors:** Taylor Follansbee, Xinzhong Dong

**Affiliations:** Department of Neuroscience, Howard Huges Medical Institute, Johns Hopkins University School of Medicine, Baltimore, MD, United States

**Keywords:** mechanical itch, alloknesis, Piezo1, Piezo2, DRG neurons, spinal cord neurons

## Abstract

Itch is a sensation in the skin which provokes the desire to scratch. In the past few decades there has been a significant elucidation of the immune and neural pathways which underly the sensation of itch. An interesting divergence in the itch pathway relates to the type of stimulation used to evoke an itchy sensation. Commonly, chemical mediators of itch such as histamine are injected into the skin where they activate their cognate receptors on sensory neurons. Another way to evoke itch, particularly in patients with chronic itch, is to use light mechanical stimulation. Investigation into these pathways utilizing the mouse model have shown that the neuronal pathways which underly chemical itch are distinct from those which mediate itch in response to mechanical stimulation. Specific populations of primary sensory neurons, spinal interneurons and transmission neurons have been identified which suggests a labeled line for itch transmission. Additionally, Piezo channels, which underly mechanosensation, were discovered to play an important role in the mechanical itch pathway. Given these novel findings relating to the mechanical itch pathway, the purpose of this review is to summarize the reports from human subjects and animal studies to highlight the advances in our understanding of mechanical itch and alloknesis.

## Introduction

The first description of mechanical itch in human subjects was made in 1909 when Titchener observed that application of weak mechanical stimulation evoked a sensation of itch (Titchener, 1909). Several decades later Bickford described an occurrence of itch sensitization in the surrounding area of skin following experience of spontaneous itching, as might occur following an insect bite. This area of skin, in which mechanical stimulation did not normally evoke a sensation of itch, had become sensitized and mechanical stimulation evoked an itch sensation (Bickford, 1938), a phenomenon later termed alloknesis (LaMotte, 1988). Alloknesis is generally measured through the presence of itch from light mechanical stimulation. In some instances following skin sensitization, alloknesis can be measured using other normally non-pruritic stimuli can be used to evoke itch such as: electrical stimulation, heat, and chemical application of citrate buffer saline or bradykinin (Ikoma et al., 2004, 2005; Hosogi et al., 2006). Given the differences, the term mechanical itch should be distinguished from alloknesis based on the presence or absence of preceding acute or pathological itch. Wherein alloknesis refers to itch evoked from a normally non itch-evoking stimuli while mechanical itch refers to itch evoked with mechanical stimulation without itch sensitization.

It was discovered that wearing a wool sweater in patients with atopic dermatitis significantly increased itch responses when compared to healthy controls (Wahlgren et al., 1991), which suggests that mechanically evoked itch worsens the pruritis experienced from patients. Intracutaneous injection of histamine into the skin evokes a sensation of itch, a wheal at the injection site, and a flare surrounding the injection site. The wheal is the result of distended skin from injection at the site while the flare response is the result of neurogenic inflammation and vasodilation caused by histamine activity on primary afferent neurons (Simone et al., 1991; Dux et al., 1996).

**Figure 1:**
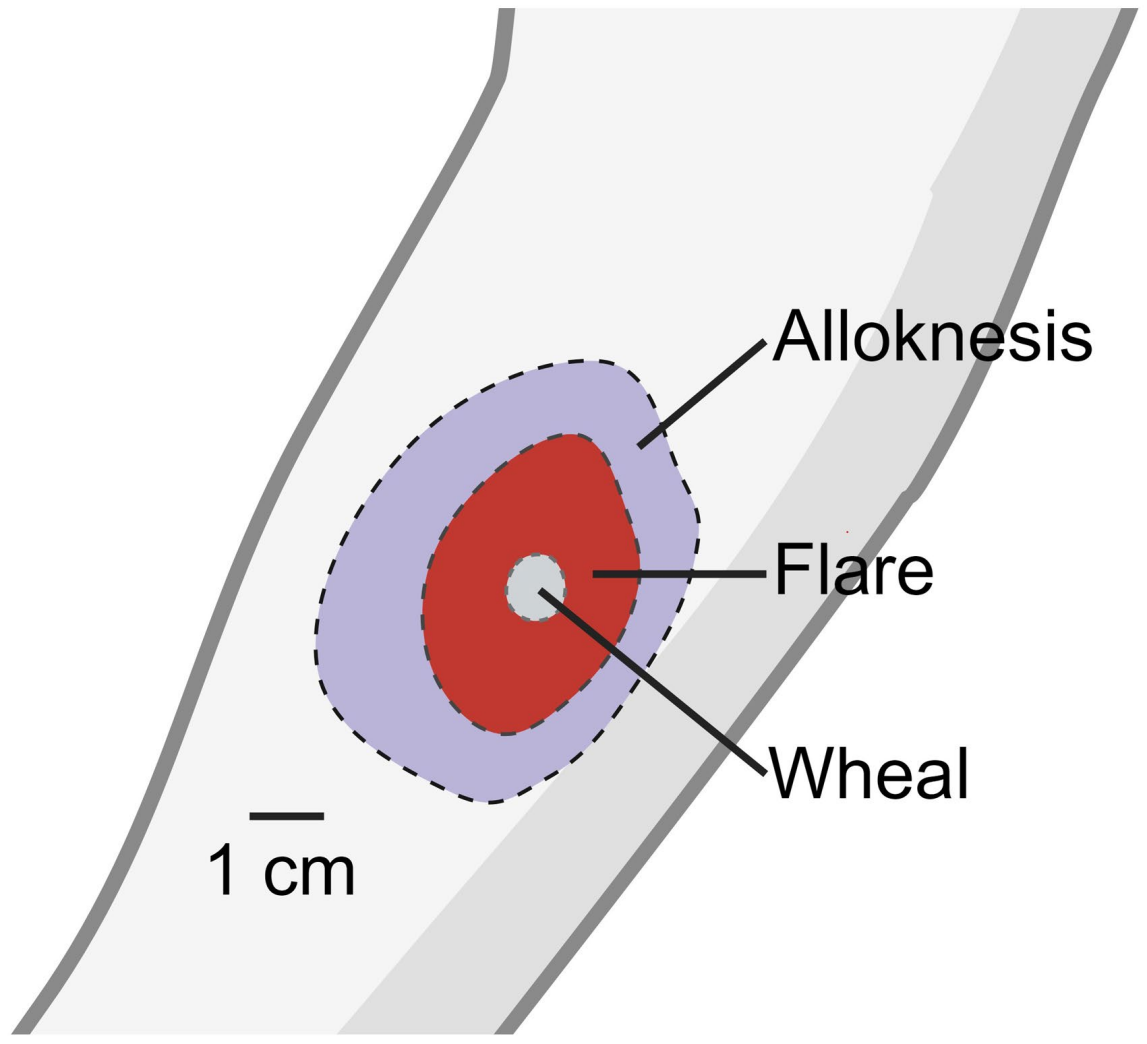
Histamine induced wheal, flare, and alloknesis.

Surrounding the site of flare reaction is an area of skin which responds to light mechanical stimulation with a sensation of itch (Simone et al., 1991; Figure 1). The spread of alloknesis was blocked with local lidocaine application and the sensation of itch and alloknesis was reduced with local cold block. Contrary to these results, when histamine was injected into the bleb of either a lidocaine like drug- chloroprocaine, or saline, the authors found that itch and alloknesis were significantly greater when injected into the site of analgesia injection (Atanassoff et al., 1999). Despite the somewhat contradictory evidence, these results suggest that the spreading of alloknesis is mediated by sensitization of neighboring sensory nerves in the skin, possibly through neurogenic inflammation. They also described an area surrounding the region of alloknesis in which noxious punctate mechanical stimulation also elicited itch which they termed hyperknesis. Clinically, some patients with dermatological maladies resulting in chronic itch, particularly atopic dermatitis, form alloknesis and hyperknesis (Wahlgren et al., 1991; Ikoma et al., 2004; Andersen et al., 2017, 2018).

## Models of mechanical itch/alloknesis

Human Models of Alloknesis and Mechanical Itch: To study alloknesis investigators have cleverly designed experiments to evoke itch from mechanical stimulation. In humans, injection of histamine produces a large area outside the flare reaction which can be mechanically stimulated with a cotton swab to evoke a sensation of itch (alloknesis) (Simone et al., 1991; Atanassoff et al., 1999). Fukuoka et al. developed a new model for mechanical itch in humans, without the necessity to inject histamine intracutaneously. In this model, a stainless iron wire is attached to a piezo actuator, allowing for modulation of swing and frequency control. Using this wire, they found that lateral movement of vellus hairs on the chin, cheek and forehead evoked intense itch sensation, without producing a pain-like component or formation of a flare (Fukuoka et al., 2013). While mechanical stimulation on the face elicited itch, stimulation elsewhere did not evoke itch, but instead produced a sensation of tickle. It is not entirely clear why mechanical stimulation to the face produced an itch sensation while stimulation elsewhere did not. It may be related to the relatively high density of vellus hairs on the face, depletion of the inhibitory spinal neurons which block mechanical itch on the face, or perhaps direct activation of c-fiber pruriceptive neurons innervating the skin between the vellus hairs (Halata, 1993; Li and Ginty, 2014).

Acute Model of Alloknesis in Mice: In the mouse, a model of alloknesis was developed which used mechanical stimulation following intradermal injection of pruritogens into the rostral back. Injection of some itch evoking chemicals like: histamine, BAM8-22, serotonin, PAR4, and cinnamaldehyde induced robust alloknesis (Akiyama et al., 2012; Domocos et al., 2020) that was assayed with light von Frey filaments (0.7 mN) applied to the rostral back of mice, approximately 7 mm from the injection site. Mechanical stimulation evoked few scratch bouts without prior injection of these pruritogens. The same group also found that injection of histamine into the skin of the calf could produce alloknesis, with the mice biting, in lieu of scratching, following mechanical stimulation (Akiyama et al., 2014). While application of mechanical stimuli does not generally evoke scratching behaviors in mice, one study shows that this changes with age and that older mice have increased mechanically evoked itch directed toward the rostral back, due to a decrease in specialized mechanosensitive cells (see below for details)(Feng et al., 2018).

Chronic Itch Models of Alloknesis in Mice: Some mouse models of alloknesis include treatment with sensitizing agents which generally produce scratching behaviors and occasionally alloknesis. These models include: the acetone ether water (AEW) model of dry skin (Akiyama et al., 2012; Feng et al., 2018; Pan et al., 2019), the squaric acid dibutylester (SADBE) model of contact sensitivity (Qu et al., 2014; Pan et al., 2019), the ovalbumin (OVA) model of atopic dermatitis (Akiyama et al., 2015), the imiquimod (IMQ) model of psoriasiform dermatitis (Follansbee et al., 2019), and the MC-903 model of atopic dermatitis (Hill et al., 2022). In these models, mice frequently present with spontaneous scratch bouts and application of mechanical stimulation evokes scratching behaviors indicative of alloknesis. Clinically, models of alloknesis are more relevant to the type of symptoms experienced by patients with chronic pruritis.

Acute Models of Mechanical Itch: Some investigators were able to reliably evoke mechanical itch in untreated mice, using light von Frey stimulation to the rostral back (Hill et al., 2022). Another group found that stimulation with light von Frey filaments to shaved skin directly rostral to the ears evoked robust mechanical itch, without prior treatment (Pan et al., 2019). Finally, ablation of spinal Npy neurons results in mice which elicit scratch bouts in response to von Frey stimulation without skin sensitization (Bourane et al., 2015).

## Receptors and primary afferent neurons for mechanical itch/alloknesis

Over the past decade significant advancements have been made in our understanding of how sensory neurons mediate chemical and mechanical itch. For chemical itch, animal studies have shown that a wide variety of pruritogens bind with the cognate receptors expressed in the free nerve endings of primary afferent neurons in the skin. These neurons are polymodal c-fibers which have recently been categorized as non-peptidergic (NP) neurons 1–3 based on unique RNA expression profiles (Usoskin et al., 2015). For example, NP1 neurons express MrgprD and respond to the pruritogen β-alanine (Liu et al., 2012), while NP2 neurons express MrgprA3 (Liu et al., 2009) and respond to chloroquine. NP3 neurons express natriuretic peptide B (Nppb) and somatostatin (Sst) which partially mediate histamine evoked itch (Solinski et al., 2019).

**Figure 2:**
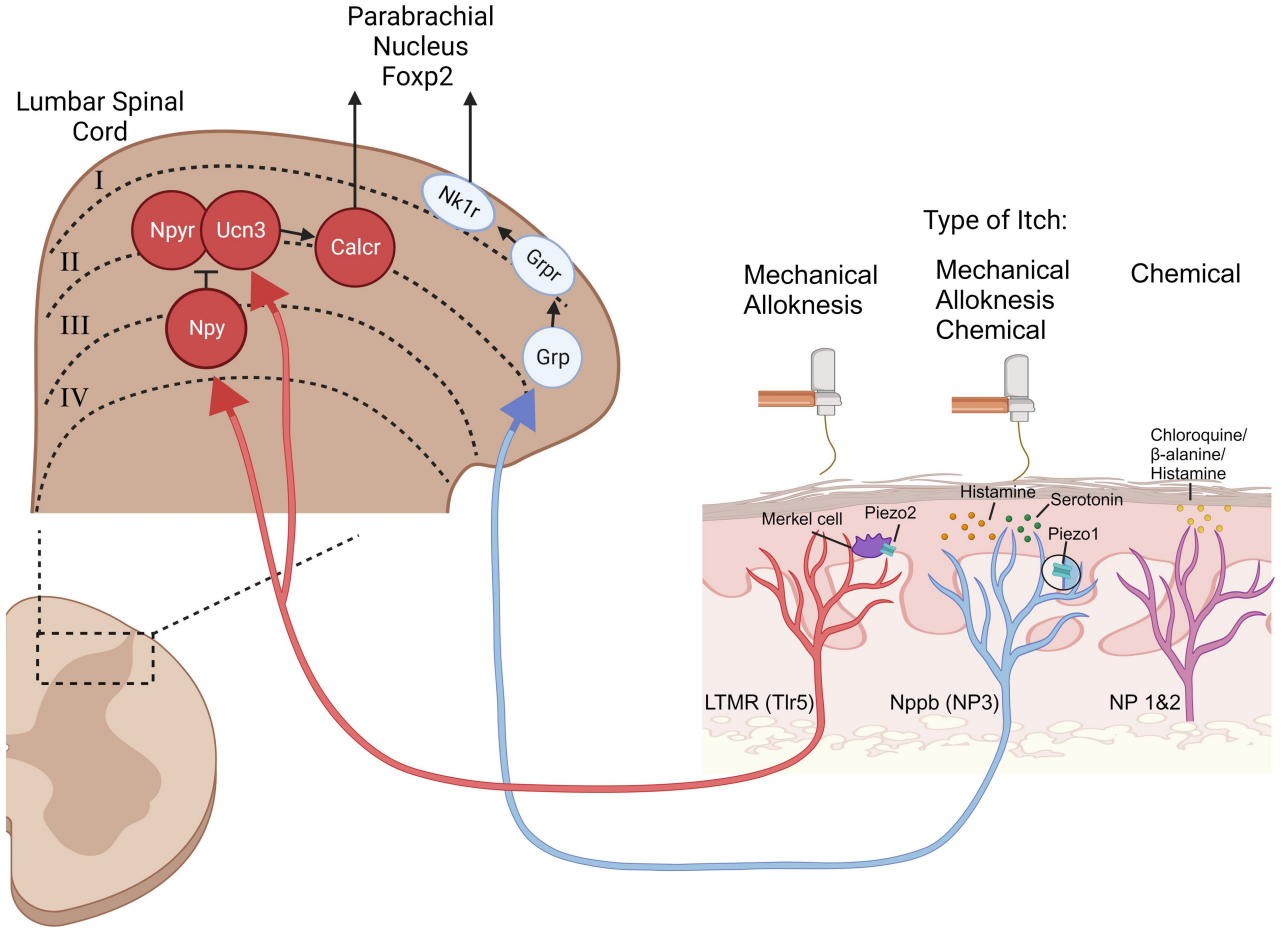
Overview of mechanical itch and alloknesis signaling pathways. The mechanical itch and alloknesis pathways (Red and Blue neurons) are distinct from the chemical itch pathways (magenta neuron). Mechanical stimulation can activate Low Threshold Mechanoreceptors (LTMRs) through piezo2 expressing Merkel cells, projecting to the spinal cord where the LTMRs synapse onto neuropeptide Y- (Npy), urocortin3- (Ucn3), and neuropeptide Y receptor- (Npyr) expressing interneurons. Npy/Ucn3 neurons synapse onto calcitonin receptor like receptor (Calcr) expressing spinoparabrachial projection neurons. Mechanical stimulation also activates piezo1-expressing natriuretic peptide B (Nppb) pruriceptive neurons which express. This pathway is likely mediated through gastin releasing peptide- (Grp), gastrin releasing peptide receptor- (Grpr), and neurokinin 1 receptor- (Nk1r) expressing neurons. Other acronyms: toll like receptor 5 (Tlr5), non-peptidergic (NP).

With respect to mechanical itch evidence determines that at least two different classes of primary afferent neurons are involved in the transmission of mechanical itch (Figure 2), both of which depend on the Piezo channels, an ion channel which opens in response to mechanical stimuli, and has well characterized roles in mechanosensation, mechanical pain, and proprioception. It was discovered that Merkel cells express Piezo2 channels, forming a synapse like connection with light touch mechanoreceptors (LTMRs). When Merkel cells were ablated or Piezo2 was knocked out in Merkel cells, alloknesis following induction of the AEW dry skin model was significantly reduced with no change in primary afferent innervation (Feng et al., 2018). Chemogenetic activation of Merkel cells suppressed alloknesis in the AEW model. Recordings from SA1 neurons in the *ex-vivo* skin-nerve preparation showed diminished SA1 firing properties in older mice and histological evidence showed fewer Merkel cells, likely contributing to age-related increases in mechanical itch sensitivity. This evidence shows that Piezo2, expressed by Merkel cells in the skin, mediates mechanical itch.

Merkel cells synapse onto LTMRs, which are a diverse class of primary afferent neurons. The LTMR population which is relevant for mechanical itch/alloknesis are the toll like receptor 5 (Tlr5) expressing population (Pan et al., 2019; Sakai and Akiyama, 2019). Blockade of these neurons using QX-314 co-injected with flagellin was sufficient to silence this population of neurons and reduce alloknesis induced from: histamine, AEW, SADBE, and IMQ. The information from Merkel cells is transmitted to the spinal cord through a population of LTMRs which express Tlr5 where they synapse onto specific itch sensitive spinal interneurons (described in the next section).

More recently, work from Ardem Patapoutian’s lab made the astonishing discovery that the NP3 (Nppb-expressing) primary pruriceptive neurons express Piezo1 and contribute to the mechanical itch pathway in a LTMR independent manner (Hill et al., 2022). Knockout of Piezo1 in all sensory neurons or more specifically in Nppb neurons significantly reduced mechanical itch. Knock-in of a constitutively active version of Piezo1 increased mechanical itch, alloknesis, and histamine evoked scratching. Injection of a Piezo1 agonist was sufficient to produce scratching behaviors without inflammation or mechanical allodynia. When they tested Piezo1 knockout mice in the MC903 model of atopic dermatitis, the development of alloknesis was reduced. These data show that there are two distinct and parallel pathways which transmit mechanical itch. Piezo2 expressing Merkel cells synapse onto LTMRs, activating a classically defined mechanical sensation pathway. Additionally, Nppb (NP3) pruriceptive neurons express Piezo1, and can transmit mechanical itch presumably through the “chemical” itch signaling pathway. It is not clear if these two mechanical itch pathways converge downstream in the spinal cord. Blockade of either pathway is sufficient to attenuate alloknesis from chronic itch models and thus both contribute to pathological itch.

## Spinal interneurons and projection neurons

Within the spinal cord, several relevant interneurons have been discovered which contribute to mechanical itch, but not chemical itch. Ablation or chemogenetic silencing of spinal neurons expressing neuropeptide Y (Npy) resulted in spontaneous scratching and touch evoked scratching behaviors (Bourane et al., 2015). Ablation of these neurons did not result in hyperknesis and did not change mechanical nor thermal pain behaviors. They found that Npy neurons receive excitatory input from peripheral LTMRs. Ablation of gastrin releasing peptide receptor (GRPR) neurons, which mediate chemical itch (Sun and Chen, 2007), did not change scratching behaviors following Npy ablation suggesting independent mechanical and chemical itch pathways. Npy neurons were found to synapse onto spinal Npy-Receptor expressing neurons. Chemogenetic activation of spinal Npyr expressing neurons increased mechanical itch, while chemogenetic silencing or ablation reduced mechanical itch (Acton et al., 2019). Perturbation of Npyr neurons did not affect chemical itch signaling. Similar to Npy-expressing neurons, Npyr-expressing neurons also receive monosynaptic input from LTMRs, with LTMRs transmitting mechanical information to both Npy- and Npyr- expressing neurons. Under normal conditions, activation of Npy neurons provides an inhibitory input onto Npyr neurons. However, if Npy neurons are silenced, the excitatory input, from LTMRs, onto Npyr neurons drives mechanical itch with no inhibition from the Npy neurons, resulting in mechanical itch behaviors. The control of Npy regulation was confirmed with pharmacological manipulation of Npyr (Gao et al., 2018).

Another class of excitatory interneurons were discovered which express urocortin 3 (Ucn3) (Pan et al., 2019). These neurons receive input from Tlr5 LTMRs as well as inhibitory input from spinal Npy neurons. Spinal ablation or chemogenetic inhibition of spinal Ucn3 neurons significantly reduced mechanical itch, while chemogenetic activation induced scratching behaviors. When Ucn3 neurons were colocalized with other markers, they found that 15% of Ucn3 neurons co-express Npyr. It is not entirely clear whether these neurons represent the same population as the Npyr neurons discussed above (Acton et al., 2019). Npy and Ucn3 co-ablation abolished the mechanical itch sensitization produced from Npy ablation alone.

It was recently discovered that there is a set of spinoparabrachial projection (SPB) neurons which relay mechanical itch but not chemical itch from the spinal cord to the parabrachial nucleus (PBN) (Ren et al., 2023). These neurons express calcitonin receptor like receptor (Calcrl) and does not colocalize with neurokinin-1 receptor (Nk1r), which is commonly expressed by the majority of SPB and spinothalamic neurons. Ablation or chemogenetic silencing of spinal Calcrl neurons at the terminals in the PBN abolished mechanical itch in Npy knockout mice. Retrograde labeling of PBN neurons showed that 18% of PBN projecting neurons express Calcl. Using a retrograde tracer injected into the PBN and cfos staining following mechanical itch and chemical itch, the authors found that the majority of mechanical itch labeled neurons co-expressed Calcrl and were located in lamina II_O_-III. Silencing either Calcr- or Nk1r-expressing neurons reduced histamine induced alloknesis (Akiyama et al., 2015; Ren et al., 2023). Calcrl-expressing SPB neurons made synapses with PBN neurons which express FoxP2. Inhibition of FoxP2-expressing neurons resulted in reduced mechanical- and chemical-evoked itch. *In vivo* calcium imaging experiments revealed that the populations of FoxP2 neurons in the PBN which respond to mechanical and chemical itch are distinct, with <3% responding to both. These results provide very good evidence for a labeled line mediating mechanical itch.

## Conclusion

In the past decade we have had several insights into the neural pathways underlying mechanical itch. From the Piezo channels underlying transmission of mechanical energy into neural signals to the spinal interneurons and spinal projection cells which relay this information to the brain. It is currently unclear how the mechanical itch pathways interact during itch sensitization to produce alloknesis. The evidence suggests that mechanical itch evoked during alloknesis depends on both parallel pathways, since ablation of either Nk1r or Calcrl neurons attenuates mechanically evoked itch in models of alloknesis. Furthermore, it appears that the pathways which underly mechanical itch are not mutually exclusive to alloknesis, since many of the neurons elucidated with the mechanical itch model also contribute to alloknesis. Clinically, more studies are needed to determine if alloknesis occurs in patients with other chronic itch maladies, and how they compare from the studies conducted on atopic dermatitis.

## Author contributions

TF: Conceptualization, Funding acquisition, Writing – original draft. XD: Conceptualization, Funding acquisition, Writing – review & editing.

## Funding

The author(s) declare financial support was received for the research, authorship, and/or publication of this article. This work is supported by NIH funding R37NS054791 and T32NS070201.

## Conflict of interest

The authors declare that the research was conducted in the absence of any commercial or financial relationships that could be construed as a potential conflict of interest.

The handling editor HH declared a past co-authorship with the author XD.

## Publisher’s note

All claims expressed in this article are solely those of the authors and do not necessarily represent those of their affiliated organizations, or those of the publisher, the editors and the reviewers. Any product that may be evaluated in this article, or claim that may be made by its manufacturer, is not guaranteed or endorsed by the publisher.

## Figures and Tables

**Table 1:** 

Itch (pruritis)	Is a sensation in the skin that provokes the desire to scratch.
Mechanical Itch	Itch evoked from mechanical stimulation without prior sensitization.
Alloknesis	Itch evoked from normally non-pruritic stimulation, such as light touch. Alloknesis generally forms as a result of skin sensitization.
Hyperknesis	Increased sensitivity of itch elicited by chemical or punctate mechanical stimulation.
